# A rare concomitant association: partial hydatidiform mole with preeclampsia without severe features, hyperthyroidism, torsion of a theca-lutein cyst and choriocarcinoma with pulmonary metastases

**DOI:** 10.1093/omcr/omaf248

**Published:** 2025-11-26

**Authors:** Maria F Peralta-Reza, Isela J Barrita-Domínguez, Alejandra Herrera-Ortiz, Aída F González-Zimbrón, Natalia M Sánchez-Solis

**Affiliations:** Department of Gynecology and Obstetrics, Hospital General Dr. Manuel Gea González, Calzada de Tlalpan 4800, Belisario Domínguez Sección 16, Tlalpan, Ciudad de México 14080, Mexico; Department of Maternal-Fetal Medicine, Hospital General Dr. Manuel Gea González, Calzada de Tlalpan 4800, Belisario Domínguez Sección 16, Tlalpan, Ciudad de México 14080, Mexico; Department of Gynecology and Obstetrics, Hospital General Dr. Manuel Gea González, Calzada de Tlalpan 4800, Belisario Domínguez Sección 16, Tlalpan, Ciudad de México 14080, Mexico; Department of Gynecology and Obstetrics, Hospital General Dr. Manuel Gea González, Calzada de Tlalpan 4800, Belisario Domínguez Sección 16, Tlalpan, Ciudad de México 14080, Mexico; Department of Hysteroscopy, Hospital General Dr. Manuel Gea González, Calzada de Tlalpan 4800, Belisario Domínguez Sección 16, Tlalpan, Ciudad de México 14080, Mexico

**Keywords:** preeclampsia, molar pregnancy, partial mole, hyperthyroidism, theca lutein cysts

## Abstract

Gestational trophoblastic disease (GTD) encompasses a spectrum of premalignant and malignant conditions arising from trophoblastic tissue. This case highlights an unusual and severe progression of a partial hydatidiform mole complicated by preeclampsia, hyperthyroidism, torsion of a theca-lutein cyst, and ultimately metastatic choriocarcinoma. The patient’s course illustrates the importance of early recognition, close monitoring, and multidisciplinary management. This rare and instructive presentation underscores the malignant potential of partial moles. Case: A 36-year-old patient who presented with abdominal pain and vaginal bleeding, associated with hypertension and a beta-human chorionic gonadotropin (β-hCG) of 1561722.6 mIU/mL. Ultrasound revealed a pattern suggestive of a partial mole. Manual vacuum aspiration (MVA) was performed, finding 750 mL of vesicular tissue. Histopathological examination confirmed a partial mole. Days later, the patient developed torsion of a theca lutein cyst, which required exploratory laparotomy. During follow-up, β-hCG levels progressively increased, indicating persistent trophoblastic disease. The patient was referred to an oncology center, where she was diagnosed with choriocarcinoma with pulmonary metastases. She is currently undergoing her first cycle of chemotherapy. The chosen regimen was etoposide and cisplatin, administered over four cycles. This decision was guided by a FIGO risk score assessment, which placed the patient in the low to intermediate-risk category. The initial response was favorable, with no observed complications during the first two cycles.

## Introduction

Gestational trophoblastic disease (GTD) encompasses premalignant conditions, such as complete and partial hydatidiform moles and malignant conditions, including invasive moles, choriocarcinoma, and placental site trophoblastic tumors. Hydatidiform moles represent 85% of cases, with partial moles frequently exhibiting a triploid karyotype [1].

The incidence of GTD varies globally, being more common in Asian and Hispanic populations. The incidence of complete mole is 1 per 1000 pregnancies, and partial mole occurs in 0.005%–0.001% of cases. Choriocarcinoma has an incidence of 1 in 40 000 to 1 in 50 000 pregnancies [2] Partial moles typically present as incomplete abortions. Theca lutein cysts are seen in 15% of molar pregnancies, typically resolving spontaneously [3] Some pregnancies progress to persistent GTD, and preeclampsia may complicate the condition in 2%–8% of cases [4] Though rare, preeclampsia can occur early in molar pregnancies [5] Upon diagnosis, a thorough evaluation is necessary to exclude conditions like preeclampsia, hyperthyroidism, and theca lutein cysts. Treatment includes evacuation, curettage, and oxytocin administration. For larger uteruses, fundal massage is recommended [6].

The definitive diagnosis is made via pathology, and patients should be monitored for postmolar trophoblastic neoplasia. The risk of persistent neoplasia is higher in complete mole (18%–28%) compared to partial mole (2%–4%) [7] Weekly hCG monitoring is required to detect persistent GTD, and chemotherapy is needed if hCG levels plateau or increase [8].

Managing these conditions requires a multidisciplinary approach, as emphasized in the review by Garner et al. [9].

## Case report

A 36-year-old patient, Mexican, homemaker. Gravida 3, para 2 (full-term deliveries in 2000 and 2003, eutocic), presents to the gynecological emergency department with complaints of colicky abdominal pain accompanied by transvaginal bleeding and high blood pressure (the record showed 140/100 mmHg). Upon initial assessment, the patient denied being pregnant. No significant personal or family medical history was reported.

Physical examination revealed symmetrical, non-congested breasts. The abdomen was globose, with a uterine fundal height of 23 cm; no fetal parts were palpable. Vaginal examination identified a posterior cervix without dilation nor evidence of bleeding. Laboratory test results showed a beta-human chorionic gonadotropin (β-hCG) level of 1561722.6 mIU/mL, total bilirubin of 1.38 mg/dL (predominantly indirect at 1.12 mg/dL), urinary creatinine 208.62 mg/dL, urinary protein of 100 mg/dL, a protein-to-creatinine ratio of 0.40, thyroid-stimulating hormone (TSH) < 0.05 μIU/mL, and free thyroxine (T4L) at 1.32 ng/dL. Other laboratory results are detailed in table 1.

**Table 1:** Laboratory tests.

07/11/2024	
b-HGC	1561722.6 mIU/mL
Leukocytes	7.4 x10´3/ul
Hemoglobin	9.95 g/dl
Hematocrit	29.34%
PLATELETS	230 x10´3/ul
Glucose	63 mg/dl
Creatinine	0.83 mg/dl
Alanine aminotransferase	31 IU/L
Aspartate aminotransferase	48 IU/L
Gamma-glutamyl transferase	28 IU/L
lactic dehydrogenase	287 IU/L
thyroid stimulating hormone	<0.05 uIU/ml
Free thyroxine	1.23 ng/dl
Prothrombin Time (PT)	11.20 seg
Partial Thromboplastin Time (PTT)	29 seg
INR	1 seg.
Urine Creatinine	208.62 mg/dl
Urinalysis: Clarity Proteins Red blood cells White blood cells Bacteria	Murky 100 mg/dl 260 c/uL ++ 4252.4 c/uL

**Figure 1:**
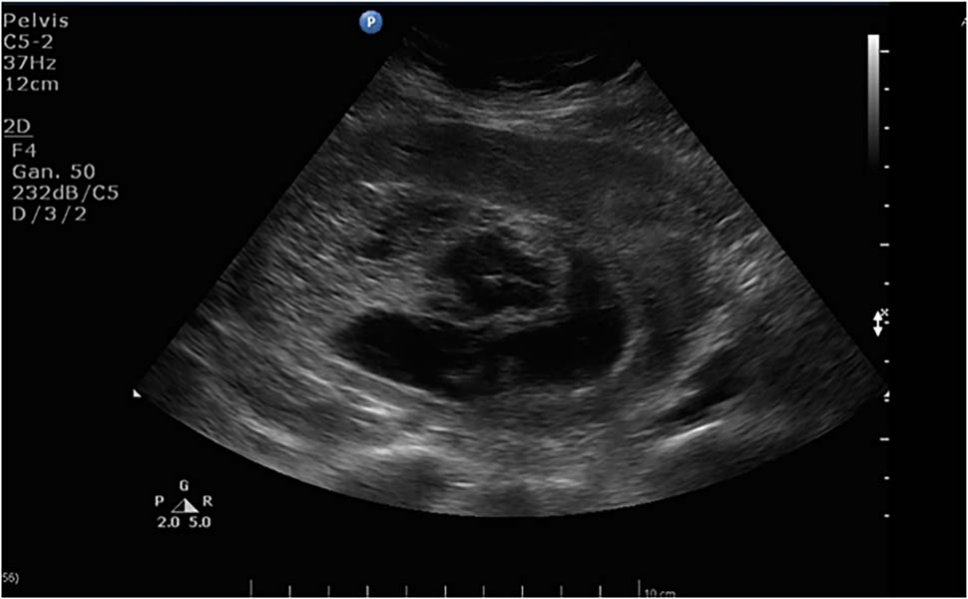
Endometrial cavity showing multiple oval anechoic images.

**Figure 2:**
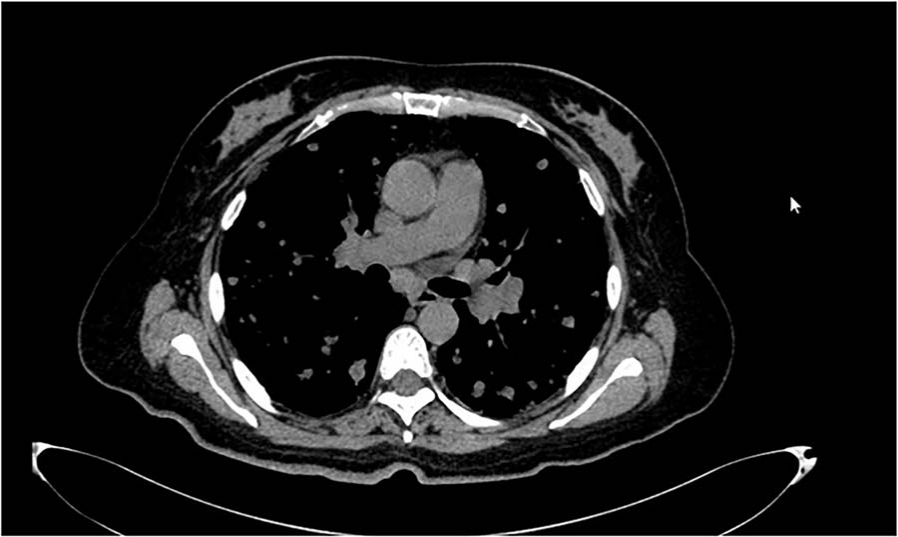
Axial thoracic CT scan showing multiple bilateral pulmonary nodules in both lower lobes, highly suggestive of hematogenous metastatic spread from gestational choriocarcinoma.

Transvaginal ultrasound demonstrated an enlarged uterus measuring 17.7x8.4x13.4 cm, with multiple anechoic oval structures within the endometrial cavity, consistent with a ‘snowstorm’ pattern; no fetus was visualized, and morphologically normal ovaries were noted. Hepatic and biliary ultrasonography showed no evidence of metastatic disease. These findings are depicted in Fig. 1. Additionally, a thoracic CT scan revealed multiple bilateral pulmonary nodules suggestive of metastatic choriocarcinoma (Fig. 2).

**Figure 3:**
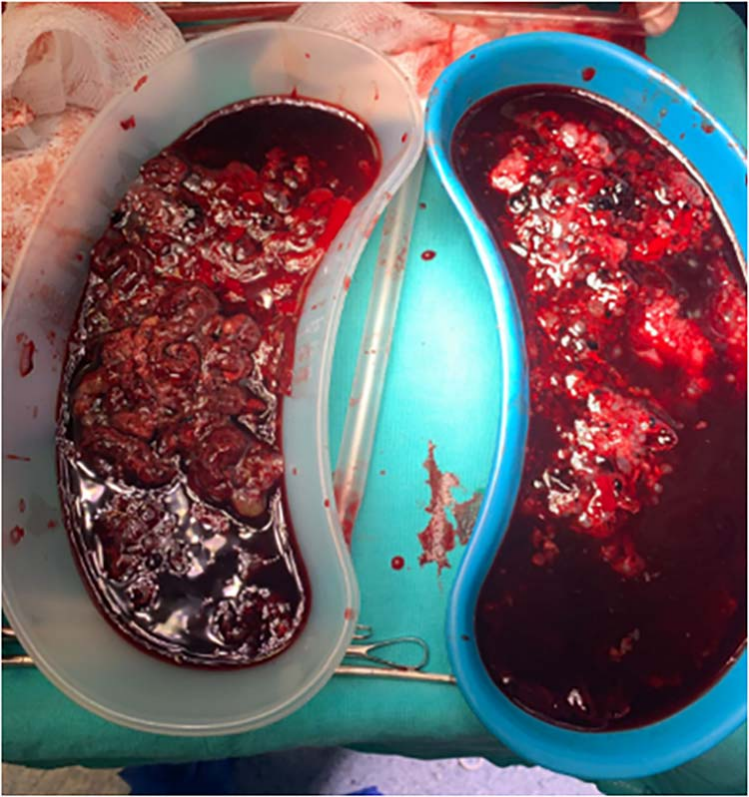
Vesicular tissue extracted during manual uterine aspiration.

The patient underwent manual uterine aspiration, yielding 750 mL of vesicular tissue with a total blood loss of 500 mL, as illustrated in Fig. 3. Histopathological analysis suggested gestational trophoblastic disease, specifically partial molar pregnancy. The patient was discharged without complications.

**Figure 4:**
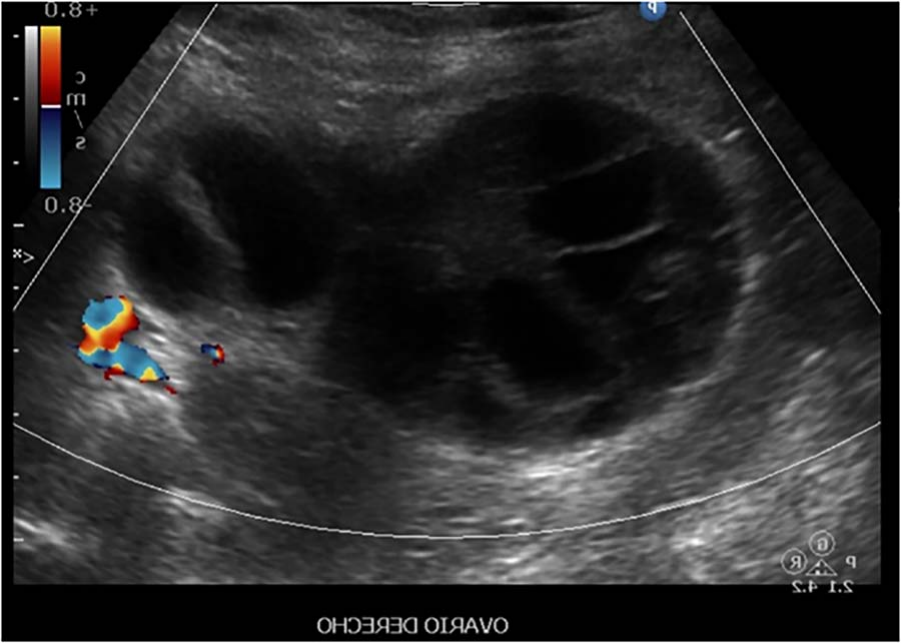
Right ovary showing a multilocular cyst without internal blood flow.

Twelve days later, the patient returned to the emergency department with a sudden onset of sharp abdominal pain localized to the right iliac fossa and radiating to the right flank, with an intensity of 8/10. An ultrasound revealed a multilocular cyst in the right ovary with sonographic signs of torsion (Fig. 4). Exploratory laparotomy identified a multilobulated cyst with two twists on its pedicle. Multiple theca lutein cysts were observed in the left adnexa and were drained, yielding moderate amounts of citrine fluid. Histopathological examination confirmed a cyst with torsion-associated changes. Subsequent follow-up of β-hCG levels revealed a plateau and subsequent increase (Table 2), raising suspicion of persistent gestational trophoblastic disease. The patient was referred to an oncology center, where she was diagnosed with choriocarcinoma with pulmonary metastases. She is currently undergoing her first cycle of chemotherapy. The chosen regimen was etoposide and cisplatin, administered over four cycles. This decision was guided by a FIGO risk score assessment, which placed the patient in the low to intermediate-risk category. The initial response was favorable, with no observed complications during the first two cycles. Diagnosis of choriocarcinoma was established based on clinical presentation, persistent elevation of β-hCG, and radiologic evidence of pulmonary metastases. Histological confirmation was not obtained due to referral and treatment initiation at the oncology center.

**Table 2:** Levels of beta fraction of human chorionic gonadotropin hormone during follow-up.

B-HGC levels	
07/11/2024	1561722.6 MIU/ML
07/13/2024	255, 266 MIU/ML
07/18/2024	34, 485 MIU/ML
07/24/2024	25, 154 MIU/ML
08/01/2024	27, 652 MIU/ML
08/08/2024	33, 450 MIU/ML
08/14/2024	33, 243 MIU/ML
08/27/2024	32, 694 MIU/ML
09/03/2024	31, 390 MIU/ML
09/10/2024	20, 380 MIU/ML
09/17/2024	22, 743 MIU/ML
09/24/2024	19, 994 MIU/ML

## Discussion

The present clinical case addresses a complex and rare condition in gynecology and obstetrics involving the convergence of multiple pathologies: partial molar pregnancy, atypical preeclampsia, hyperthyroidism, thecal-lutein cyst torsion, and choriocarcinoma with pulmonary metastasis, which highlights the importance of early diagnostic approaches, multidisciplinary management, and prolonged follow-up.

Partial molar pregnancy is a form of gestational trophoblastic disease (GTD) characterized by abnormal trophoblast proliferation and typically triploid karyotype. Although less aggressive than complete molar pregnancy, it may be associated with complications such as gestational hypertension, hyperthyroidism, and, in some cases, malignant choriocarcinoma. Proper uterine evacuation and follow-up with serum hCG levels are essential to rule out persistent or metastatic disease [1].

Preeclampsia associated with partial molar pregnancy is less frequent than in complete molar pregnancy but may manifest early due to the trophoblast’s role on maternal angiogenic mechanisms. While no severe features were detected in this case, close monitoring is critical since these patients have a higher risk of progression to severe and potentially fatal conditions [2].

Hyperthyroidism in GTD is a well-documented complication caused by the thyrotropic activity of human chorionic gonadotropin (hCG), which can mimic thyroid-stimulating hormone (TSH). This hyperthyroidism is transient and usually resolves after uterine evacuation, but careful management is required during the acute phase to prevent thyroid storm [3].

Thecal-lutein cysts are common in molar pregnancies due to ovarian stimulation by elevated hCG levels. Torsion of these cysts is a gynecological surgical emergency, as demonstrated in this case. Timely intervention prevents additional complications such as ovarian necrosis or intraperitoneal hemorrhage [4].

Metastatic choriocarcinoma is a rare but severe complication of GTD, more common in patients with complete molar pregnancy, although it can also occur with partial molar pregnancy. Pulmonary metastases are the most frequent and, in this case, highlight the importance of strict follow-up using hCG levels and imaging studies for staging and monitoring [5].

Comprehensive management for this patient includes:

– Uterine evacuation: Initial procedure to remove the molar tissue.
– Monitoring beta-hCG levels: Weekly until normalization.
– Hyperthyroidism control: Using antithyroid medications and hormonal monitoring.
– Pulmonary monitoring: Periodic imaging to rule out metastatic progression.
– Chemotherapy: Indicated for metastatic choriocarcinoma; regimens include methotrexate or EMA-CO (etoposide, methotrexate, actinomycin D, cyclophosphamide, and vincristine) based on risk (FIGO, 2021).

This collaborative approach greatly enhances prognosis and allows for comprehensive management of complications.

## Consent

Written informed consent was obtained using the official OMCR form. The patient gave explicit authorization for the publication of this case, including the use of anonymized clinical images and relevant medical data.
